# Oral health status and need for oral care of care-dependent indwelling elderly: from admission to death

**DOI:** 10.1007/s00784-016-2011-0

**Published:** 2016-11-28

**Authors:** Arie R. Hoeksema, Lilian L. Peters, Gerry M. Raghoebar, Henny J. A. Meijer, Arjan Vissink, Anita Visser

**Affiliations:** 1 Department of Oral and Maxillofacial Surgery, BB70, University of Groningen, University Medical Center Groningen, PO Box 30.001, NL-9700 RB Groningen, the Netherlands; 2 Oral Health Care Center ‘Mondzorgcentrum Winschoten’, Winschoten, the Netherlands; 3 Department of Oral Function and Prosthetic Dentistry, Dental School, University of Groningen, University Medical Center Groningen, Groningen, the Netherlands

**Keywords:** Care-dependent, Elderly, Oral health status, Cooperation, Nursing home, Dental implants

## Abstract

**Objectives:**

The objective of this study is to assess oral health and oral status of elderly patients newly admitted to a nursing home from admission until death.

**Materials and methods:**

Oral health, oral status, need for dental care, cooperation with dental treatment, and given dental care were assessed by two geriatric dentists in all new long-stay patients (*n* = 725) admitted to a nursing home between January 2009 and December 2013. All patients were followed from admission until death or until they left the nursing home.

**Results:**

At admission, dementia patients were significantly older than somatic patients; median [IQR] ages were, respectively, 85 [79–89] and 81 [76–87] (*p* = 0.001). In addition, edentulous patients were significantly older than patients with remaining teeth, 83 [79–89] versus 80 [74–86] (*p* = 0.001) years. Thirty percent of the admitted patients died within 12 months after admission. A small minority (20%) of the patients had their own teeth. In this group, poor oral hygiene (72%), caries (70%), and broken teeth (62%) were frequently observed. Edentulous patients were significantly more cooperative with treatment than patients with remaining teeth (64 versus 27%). Finally, significantly less professional dental care was given to edentulous patients when compared to patients with remaining teeth (median 90 [IQR 60–180] versus 165 [75–375] min).

**Conclusion:**

When compared to edentulous elderly patients, patients with remaining teeth were younger at admittance, were more often non-cooperative, and had a poorer oral health and higher need for dental care.

**Clinical relevance:**

It is important that health care workers ensure adequate oral health and dental care to frail elderly, especially for elderly with remaining teeth.

## Introduction

The increasing life expectancy and decreasing birth rate, particularly in the industrialized countries, have resulted in a progressive demographic transformation of the society into a society characterized by an increased proportion of elderly [1]. In 2020, about 40% of the population in the northern part of the Netherlands, the region where this study was performed, will be over 65 years of age (CBS 2011) [2]. Recently, this area was identified as having optimal circumstances for research into healthy aging [3]. The number of people over 80 years in this area will increase to approximately 10% of the population during the next three decades.

When elderly become care-dependent, their oral health usually becomes worse and gets less attention [4]. Oral health is neglected partly because care-dependent elderly need care on many levels, and this interferes with their activities of daily living such as food intake, drug intake, getting dressed, bathing, general health care, and physiotherapy. As a result, less time is reserved for activities that are commonly considered less important by elderly, including oral care [5, 6]. The lack of attention for oral care is a hidden health hazard as dental awareness and oral health are thought to be essential for general health and quality of life [7, 8]. A decline in the number and quality of natural teeth, a phenomenon that is quite common among elderly, can cause malnutrition, pain, and severe functional and esthetic problems, which in turn have a strong effect on oral health-related quality of life [8] and daily activities [9]. Dental and periodontal diseases are also significantly associated with occurrence and disease activity of diabetes [10], cardiovascular disease [11], atherosclerosis [12, 13], rheumatoid arthritis [14], kidney function [15], pneumonia [16], multiple sclerosis, and other systemic immune problems [7]. Furthermore, cognitive impairment and accumulation of amyloid plaques was shown to be more prevalent in persons with chewing difficulties [17] and poor oral health [18]. The impact of oral health on general health is probably even more severe in institutionalized elderly; this group is known to have higher levels of *Candida* species and *Staphylococci* in their oral environment, leading to a high risk of opportunistic infections [19].

Due to this increasing need for dental services for elderly, research is needed to accurately characterize the oral health status and needs of the growing number of homebound and institutionalized elderly [12]. Several cross-sectional studies have reported on the oral health status of indwelling elderly [20–22], but data on oral health status at admission and the specific problems and needs that occur during their stay in a nursing home are sparsely reported in the literature. Therefore, the aim of this study was to assess the oral health and oral status of long-stay elderly newly admitted to a nursing home, from admission until death or until they left the nursing home. The focus was on the specific needs of elderly with remaining teeth compared to edentulous elderly and their cooperation with dental treatment.

## Patients and methods

### Participants

All new elderly, 65 years and older referred to the long-stay somatic or dementia departments of two regional operating large nursing homes in the north of the Netherlands between January 2009 and December 2013 were scheduled for a standardized dental screening within 6 weeks after admission. Standardized dental screening was care as usual in both nursing homes and was performed according to the guidelines for oral care for elderly in nursing homes [23]. For all patients/residents in Dutch nursing homes, the costs for dental screening and dental care are provided by the national health insurance. Demographics, oral health status, the need for dental care, mortality, and cooperation of the elderly patients for dental treatment were scored. All patients were followed until December 31, 2014 unless they deceased before. Verbal consent was obtained from either the subject or their legal representatives. The ethics review board of the University Medical Center Groningen provided a waiver that this is not an experimental study with tests subjects as stated in the Medical Research Involving Human Subject Act as the dental screening and oral care was part of the care as usual (Letter M13.133088).

### Data collection

Standardized dental screening, as described above, was performed by ARH and AV, both geriatric dentists. These dentists have worked together for over 15 years, and were very experienced in performing oral examinations in geriatric patients. All patients were seen for a first dental screening within the first 6 weeks after admittance to the nursing home according to the guidelines of oral care for nursing homes in the Netherlands [23]. Verbal consent for this screening was obtained from all patients. In case dementia patients could not agree themselves as a result of their cognitive impairment, consent of the family of the patients helped out to let the dentist inspect the mouth as oral care is considered to be a part of routine daily care. As a result, all patients were screened. The first dental screening was performed in the patient’s own room in the nursing home as most residents were very immobile or severely cognitive impaired. Basic dental instruments were used like mirrors and if appropriate dental probes in combination with professional bright flashlights. In case elderly had remaining teeth, a second thoroughly dental screening was performed in the dental office of the nursing home if possible.

**Fig. 1:**
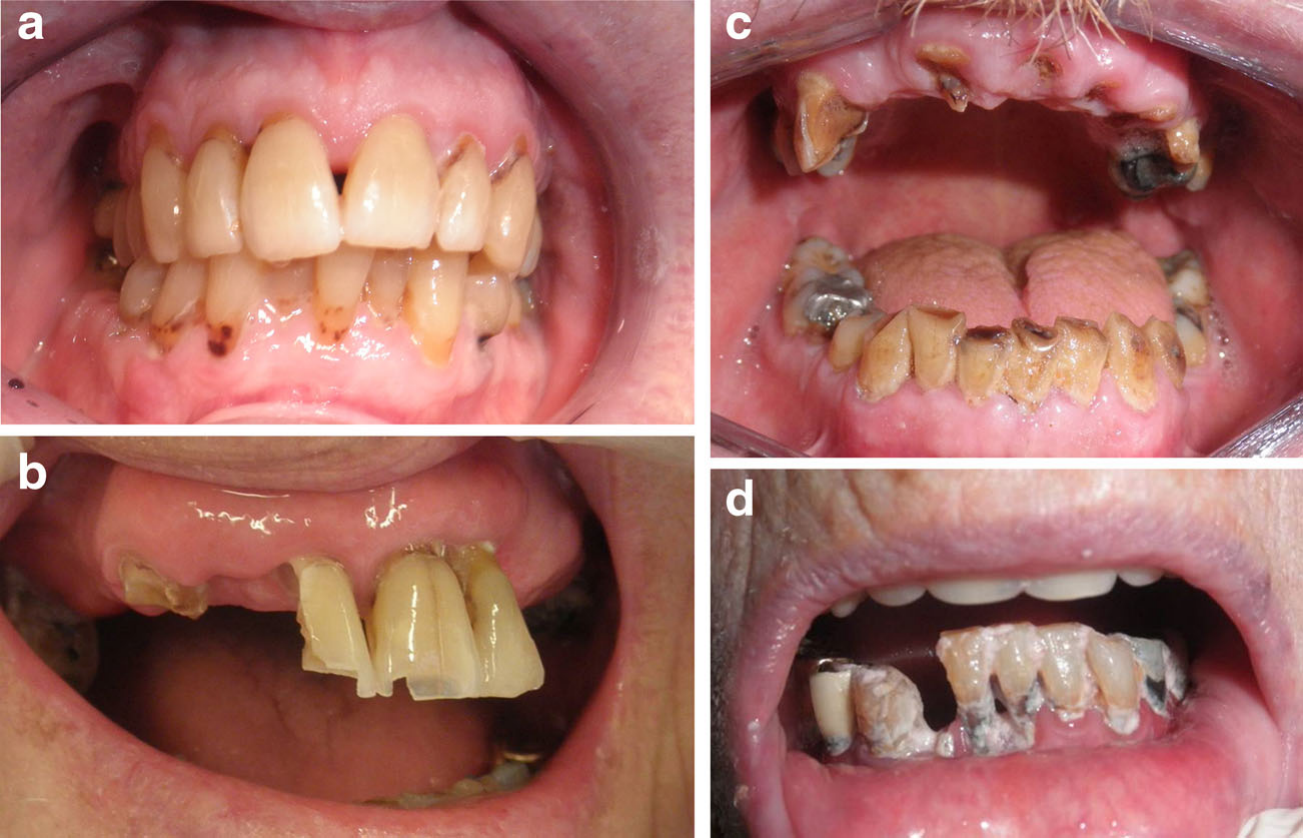
Oral hygiene (plaque) scores, modified after Mombelli et al. [24]. **a** Score 0 plaque. **b** Score 1 plaque. **c** Score 2 plaque. **d** Score 3 plaque

Besides demographic characteristics of the patients, the following items were scored and noted on a standardized intake form during the dental screening:

- Presence or absence of natural teeth. Patients were categorized as “patients with remaining teeth” if at least one natural tooth was present in the mouth. Edentulous patients (i.e., patients without teeth) with one or more residual roots underneath their fully dentures were also considered as edentulous.
- Cooperation of the patient with dental examination and treatment (cooperative or non-cooperative). If a patient suffering from dementia firmly resisted (self-defense behavior, kicking, shouting, etc.) during inspection of the oral cavity or during simple necessary daily oral care (e.g., tooth brushing, denture cleaning), the patient was considered to be non-cooperative.
- Oral hygiene was rated as good in the absence of visual plaque according to the score of Mombelli et al. [24] (score 0; Fig. 1a), reasonable when some plaque was detected (score 1; Fig. 1b), poor when thin layers of plaque were seen on all surfaces (score 2; Fig. 1c), and bad when layers of plaque were present in the whole dentition **(**score 3; Fig. 1d**)**.
- Track record of provided treatments. Of all dental treatments, the treatment time was recorded in tracks of 5 min.

On the basis of the screening data, the patients were categorized into the following two groups: patients with remaining teeth and edentulous patients. The latter group included patients with or without full dentures and patients with overdentures attached to dental implants. Next, a standardized specific group-related screening was done to collect additional information about their dental situation.

#### Patients with remaining teeth (including single-tooth replacement on implants)

- Number of teeth present;
- Presence of partial prostheses (yes or no);
- Number of decayed and broken teeth;
- Presence of dental implants (number, brand, and type of suprastructure).

As a reliable full periodontal screening was not possible in most residents due to lack of cooperation, such data were not entered in our analyses for remaining teeth and/or implants. Visual inspection and basic dental instruments were used to detect caries. No X-rays were used as this was not available in the nursing homes and most patients were not able to cooperate enough to make good X-rays. When decay was detectable with the human eye and/or with the use of dental probes (in other words, when it was through the enamel and in the dentine), it was considered as caries.

#### Edentulous patients

- Fitting and retention of prosthesis (good, moderate, bad);
- Occlusion (adequate contact between the lower and upper denture);
- Broken denture teeth;
- Fractured denture base;
- Presence of dental implants to retain implant-retained dental prostheses. When dental implants were present, number and brand of the dental implants were noted as well as the type of suprastructure (e.g., ball attachments, bar-clip construction).

As reliable probing of the peri-implant tissue was not possible in most residents due to lack of cooperation, such data were not entered in our analyses for implants.

After the standardized dental screening at intake, a treatment plan was made for all patients, who then received the dental care they needed while taking their health status in account. For example, dental treatment was not always possible and desirable due to severe health problems, poor mobility, and personal wishes. Only rather simple treatments were performed such as recall, cleansing, extractions, dental restorations and relining, and rebasing or renewal of dentures, but no complex prosthodontics were used such as fixed partial dentures (crown and bridgework). The number of consultations and the given dental treatment (in minutes) were scored from date of admission until the resident died or left the nursing home.

### Statistical analysis

Baseline characteristics were analyzed using descriptive statistics. Differences between elderly subgroups that differed on individual characteristics (i.e., oral health status, age, gender) were calculated with Kruskal-Wallis tests and Pearson’s chi-squared tests, where appropriate. A *p* value of ≤0.05 was considered as statistically significant. All statistical analyses were performed with SPSS Statistics 22.0 (SPSS Inc., Chicago, IL).

## Results

### Patients

**Fig. 2:**
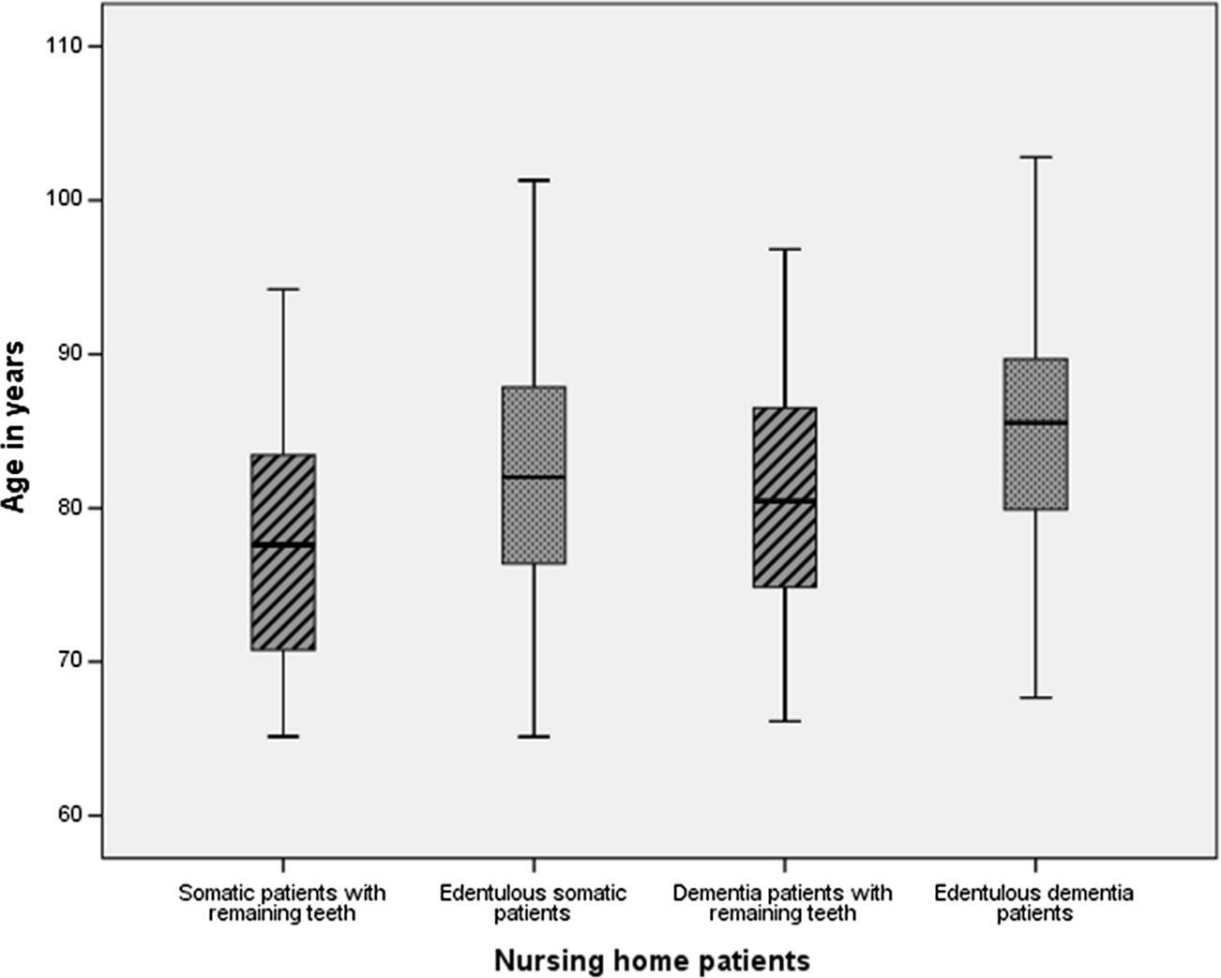
Box plot of the differences in age (median, IQR) at admission between somatic patients with and without remaining teeth when compared to dementia patients with and without remaining teeth. Dementia patients were significantly older than somatic patients (*p* = 0.001). Edentulous patients were significantly older than patients with remaining teeth (*p* = 0.001)

Between January 2009 and December 2013, 725 patients (479 dementia patients, 246 somatic patients) were examined for dental screening within 6 weeks after admission to the nursing home (Table 1). The median age at admission was 83 years [IQR 78–88]. Dementia patients were significantly older than somatic patients (*p* = 0.001). In addition, edentulous patients were significantly older than patients with remaining teeth (*p* = 0.001; see Fig. 2). During follow-up, 27% (*n* = 198) of the patients remained institutionalized and 15% (*n* = 107) of the patients left the nursing home to live either in another nursing home in the vicinity of the family or went back home to be nursed there or to die near their family. Almost 60% (*n* = 420) of the patients died during the follow-up period, of whom 29% within the first year after admission (*n* = 208) (Table 2).

**Table 1:** Characteristics of the patients

Year of admission	2009	2010	2011	2012	2013	Total
New admissions	123	147	144	149	162	725
Demographics						
Age (median, IQR)	83 [76–88]	83 [77–88]	84 [78–89]	85 [80–90]	83 [78–88]	83 [78–88]
Gender (female, %)	75 (61)	90 (61)	86 (60)	94 (63)	102 (63)	447 (62)
Nursing status						
Dementia (*n*, %)	83 (67)	95 (65)	107 (74)	115 (77)	79 (49)	479 (66)
Somatic (*n*, %)	40 (33)	52 (35)	37 (26)	34 (23)	82 (51)	246 (34)
Oral status						
Dentulous (*n*, %)	26 (21)	31 (21)	33 (23)	29 (19)	33 (20)	152 (21)
- Dentulous with implants (*n*, %)	0 (0)	0 (0)	0 (0)	0 (0)	0 (0)	0 (0)
Edentulous (*n*, %)	93 (76)	113 (77)	108 (75)	119 (80)	123 (76)	556 (77)
- Edentulous with implants (*n*, %)	4 (3)	3 ( 2)	3 (2)	1 (1)	6 (4)	17 (2)

**Table 2:** Overview of patients per cohort of admission year who stayed alive and died after admission or left the institution

Year of admission		2009	2010	2011	2012	2013	Total
New admissions		123	147	144	149	162	725
Died during study period	*n* (%)	85 (69)	107 (72)	97 (67)	74 (50)	57 (35)	420 (58)
Left nursing home	*n* (%)	26 (21)	23 (16)	13 (10)	21 (15)	24 (15)	107 (15)
Still alive at end of study	*n* (%)	12 (10)	17 (11)	34 (24)	54 (36)	81 (50)	198 (27)
Died in first year of admission	*n* (%)	32 (26)	55 (37)	39 (27)	36 (24)	46 (28)	208 (29)
Duration of stay (in months) in the nursing home until death of patients who died within the first year after admission (median, IQR)		3.8 [2.9–5.8]	3.8 [2.3–5.9]	3.7 [1.8–8.1]	5.6 [3.4–7.6]	3.1 [1.1–6.0]	3.9 [2.2–6.9]

The oral health status and age of the patients who died in the first year of admission did not differ significantly from those who stayed for a longer period of time in the nursing home (*p* = 0.81 and 0.66, respectively). The years before admission, hardly any of the patients had visited their family dentist. Many patients or their representatives could not even identify their former family dentist. Thus, except for a few patients, no information about former dental treatment was available.

### Oral status

About 20% of the admitted patients had remaining teeth. Patients with remaining teeth were far more often non-cooperative than edentulous patients (64 versus 27%, *p* = 0.001; Table 3 **)**. Cooperation did not differ between males and females.

**Table 3:** Oral status of patients with remaining teeth or dental prostheses at admission to the nursing home

Year of admission	2009	2010	2011	2012	2013	Total
Patients with remaining teeth (*n*)	26 (21%)	31 (21%)	33 (23%)	29 (19%)	33 (20%)	152
Non-cooperative, *n* (%)	17 (65)	23 (74)	17 (51)	21 (72)	20 (60)	98 (64)
Poor oral hygiene, *n* (%)	20 (75)	22 (70)	23 (70)	22 (76)	23 (70)	110 (72)
Caries (≥1), *n* (%)	20 (75)	20 (65)	22 (67)	22 (76)	23 (70)	107 (70)
Broken teeth (≥1), *n* (%)	20 (75)	19 (61)	19 (58)	19 (66)	17 (51)	94 (62)
Dental treatment time during admission (in minutes; median, IQR) (diseased patients)	195 [75–735]	150 [60–195]	225 [120–375]	^a^	^a^	165 [75–375]
Patient with dental prostheses (*n*)	97 (79%)	116 (79%)	111 (77%)	120 (81%)	129 (80%)	573
Non-cooperative, *n* (%)	34 (35)	42 (36)	23 (20)	26 (21)	29 (22)	154 (27)
Poor fitting prosthesis, *n* (%)	31 (32)	40 (34)	40 (36)	31 (26)	28 (22)	170 (30)
Poor retention, *n* (%)	33 (34)	41 (35)	37 (33)	39 (33)	29 (23)	179 (31)
Non-functional occlusion, *n* (%)	42 (43)	54 (47)	33 (30)	26 (22)	30 (23)	185 (32)
Dental treatment time during admission (in minutes; median, IQR) (concerns diseased patients)	120 [60–225]	75 [45–135]	90 [60–165]	^a^	^a^	90 [60–180]

^a^Percentage of patients still alive too high for valid analyses

### Patients with remaining teeth (*n* = 152)

More males than females had remaining teeth (25 versus 19%, *p* = 0.05). Overall, oral hygiene was poor at admission (72%) and stayed poor during the stay in the nursing home. Caries was seen frequently (70% of the patients had more than one caries lesion). Furthermore, 62% of the patients had one or more broken teeth or broken restorations (Table 3). None of the patients had dental implants retain a dental crown.

Patients with remaining teeth needed significantly more dental treatment time during their stay in the nursing home than edentulous residents (median 165 min [IQR 75–375] versus 90 [IQR 60–180], *p* = 0.001). Three categories of patients with remaining teeth could be distinguished:

1. Patients with a dentition in no need of or minor need for dental treatment (fabrication of one or more dental restorations and/or fabrication or repair of partial dentures; 13%). Oral care was aimed to sustain the maximal possible oral function;
2. Patients with a dentition in need of excessive dental care, such as extractions combined with multiple restorations and/or the fabrication of partial prostheses (74%). Oral care was aimed to uphold minimal oral functioning;
3. Patients in need of removal of all remaining teeth (to sustain or improve general health) combined with prosthodontic rehabilitation (if applicable and possible) after removal of the teeth (13%). In the great majority of this group of patients, it was not possible to fabricate a functional denture, mainly because residents were very uncooperative, could not get used to wearing a denture, or refused to wear a denture.

### Edentulous patients (*n* = 556)

At admission, 89% of the edentulous patients had a set of full dentures. From the patients who had a denture, 7% did not wear their dentures, 6% only wore upper dentures, and 0.3% only lower dentures. For those who wore their dentures, in about one third of the patients, the fit and retention of the prostheses at intake was poor to very poor. A new set of dentures was made in one third of the patients who came in without prosthesis, but the fabrication of this new set of dentures was only successful in 66% of these cases, as one third were no longer able to get used to a new set of dentures.

Among the patients with dental implants (*n* = 17), different types and brands of implants and suprastructures were observed to retain a mandibular denture (Table 4). In two out of the five implant patients who did not wear their implant-retained mandibular denture anymore, the suprastructures had to be removed due to mucosal trauma caused by the suprastructure. Severe peri-implant bone loss with massive bleeding and discharge of pus on probing were present in one patient suffering from dementia. In this patient, the transmandibular implant (TMI) had to be removed under general anesthesia, as described by Visser et al. [21].

**Table 4:** Oral status of edentulous patients with implants at admission nursing home

	Patients, *n* = 17 (%)
Cooperation	
Non-cooperative, *n* (%)	6 (35)
Oral hygiene	
Unsatisfactory, *n* (%)	5 (29)
Type of implant/suprastructure	
Ball attachments, *n* (%)	5 (29)
Bar, *n* (%)	9 (53)
Transmandibular implant, *n* (%)	3 (18)
Number of implants	
Two implants, *n* (%)	13 (76)
Four implants, *n* (%)	4 (24)
Fit of prosthesis	
Insufficient (score ≥1), *n* (%)	2 (12)
Wearing prosthesis	
Number, *n* (%)	3 (18)

## Discussion

Analysis of the oral health and oral status of newly admitted long-stay elderly revealed that at admission, age of patients with remaining teeth was lower than of edentulous patients as well as that dementia patients were older than somatic patients. With respect to the specific needs of elderly with remaining teeth, oral health and oral hygiene of these patients were usually very poor and their need for oral care was high. In this respect, it is important to note that patients with remaining teeth, often patients that are in need for dental treatment, are less cooperative for dental treatment when compared to edentulous patients.

The proportion of elderly with remaining teeth in our patient cohort was rather small when compared to edentulous elderly, which may be related to the rather high age of admission of patients to the nursing home. The number of patients with remaining teeth will probably increase during the next decades as the percentage of elderly with remaining teeth is rapidly growing. Currently, over the age of 75 years, edentulous patients are still in the far majority in the northern part of the Netherlands [25], while in the 65–75-year-old elderly, the number of edentulous patients is rapidly declining when compared with elderly of 65–75 in earlier years [26]. This foreseen increase in number of elderly with remaining teeth will have significant impact on the dental needs of institutionalized elderly. The organization of oral care in nursing homes is therefore in high need of optimization as many of the institutionalized elderly are not able to take care for their teeth themselves and their dental awareness is low.

According to the literature, oral health of elderly is often poor [5, 22, 27, 28]. Most of these data were retrieved from studies looking at cohorts of elderly patients living in nursery or elderly homes for many years. Moreover, these studies claim that oral health is becoming poor when elderly are nursed in the nursing home due to the lack of oral self-care and the neglect and shortage of time among caregivers to provide their residents with the oral care they need [22, 29]. We addressed the level of oral care and oral health at admission to the nursing home and showed that oral health as well as oral care was already poor in most of the cases when admitted to the nursing home. The cross-sectional study of Gerritsen et al. [22] described the oral health of all indwelling residents residing in a nursing home at a certain moment, thus irrespective of how long these elderly already lived in that nursing home. By contrast, we reported the oral health status of all newly admitted residents that were admitted to the studied nursing homes during a period of 5 years. We also showed, as mentioned before, that many of these elderly patients are not very cooperative with oral care. We hypothesize that this poor oral health at admission may be due in part to the fact that subjects who become care-dependent commonly stay in their own homes with the aid of day care nurses visiting their homes as long as possible. Moreover, most of these subjects are not able anymore to visit a dentist due to transportation or mobility problems and have often impaired skills to clean their dentition or dentures in a proper way. In other words, mobility problems are probably strong factors promoting dental decline, while elderly themselves are often not aware of these, usually rather slowly progressing, changes in their lives, reflected in, among others a worse oral hygiene and declining dental treatment by missing regular dental checkups due to general health problems. In this respect, it is important to note that most home care nurses are untrained in providing oral hygiene care [30]. The same counts for nurses in nursing homes [31]. In addition, multi-drug treatment, which is quite common in elderly, is often accompanied by xerostomia and reduced salivary flow. This impairs the self-cleansing of the mouth and promotes dental decay [32, 33].

Improved awareness of the poor oral health status of the elderly should be an urgent priority among care providers. When elderly start becoming care-dependent, care workers should already keep an eye on their dental status and ensure that life-proof dental care is given to these elderly, either by themselves or by actively asking support from dental professionals. In other words, there is an increasing need for geriatricians to become aware of the health hazard of poor oral health and for dentists to have training in geriatrics [34]. If oral health care providers and geriatricians do not take the responsibility of persuading society of the importance of adequate oral health, weakened oral health of community-dwelling older people will become a new geriatric syndrome [35].

Patients with remaining teeth were far more often non-cooperative and agitated than patients without remaining teeth. They were often difficult to treat, even in experienced hands. Patients with dementia can be very agitated and otherwise difficult to treat. [36]. Such patients can be in pain but are often not able to communicate that they are in pain, resulting in non-cooperative behavior in daily, medical and oral care. In our clinic, behavior of patients, appointed by nursing staff, often improved after treating possible pain complaints, which is in agreement with the findings of Husebo et al. [37], who showed that pain was related to agitation. In addition, patients are often also in very poor general condition; almost one third of the patients died during the first year of admittance. This poor general health makes dental treatment even more complex or sometimes impossible as these patients are already in a very poor health or mental condition at admittance.

## Conclusions

When compared to edentulous elderly patients, patients with remaining teeth were on average younger at admittance, were more often non-cooperative, and had a poorer oral health and higher need for dental care.

## References

[1] Branca S, Bennati E, Ferlito L, Spallina G, Cardillo E, Malaguarnera M, Motta M (2009). The health-care in the extreme longevity. Arch Gerontol Geriatr.

[2] Garssen J (2011) Demografie van de vergrijzing. CBS 2011 (Dutch central agency for statistics). http://www.cbs.nl/NR/rdonlyres/D7D8F678-F22B-445F-8A6F-A635D376A344/0/2011demografievandevergrijzingart.pdf Accessed April 26, 2013

[3] Klijs B, Scholtens S, Mandemakers JJ, Snieder H, Stolk RP, Smidt N (2015) Representativeness of the LifeLines cohort study. PLoS One 10. doi:10.1371/journal.pone.0137203 10.1371/journal.pone.0137203 PMC4557968 26333164

[4] Panchbhai AS (2012). Oral health care needs in the dependent elderly in India. Indian J Palliat Care.

[5] Jokstad A, Ambjørnsen E, Eide KE (1996). Oral health in institutionalized elderly people in 1993 compared with in 1980. Acta Odontol Scand.

[6] Cohen Mansfield J, Lipson S (2002). The underdetection of pain of dental etiology in persons with dementia. Am J Alzheimers Dis Other Demen.

[7] Somma F, Castagnola R, Bollino D, Marigo L (2010). Oral inflammatory process and general health. Part 1: the focal infection and the oral inflammatory lesion. Eur Rev Med Pharmacol Sci.

[8] Tramini P, Montal S, Valcarcel J (2007). Tooth loss and associated factors in long-term institutionalised elderly patients. Gerodontology.

[9] Genkai S, Kikutani T, Suzuki R, Tamura F, Yamashita Y, Yoshida M (2015). Loss of occlusal support affects the decline in activities of daily living in elderly people receiving home care. J Prosthodont Res.

[10] Teeuw WJ, Gerdes VE, Loos BG (2010). Effect of periodontal treatment on glycemic control of diabetic patients: a systematic review and meta-analysis. Diabetes Care.

[11] Janket SJ, Baird AE, Chuang SK, Jones JA (2003). Meta-analysis of periodontal disease and risk of coronary heart disease and stroke. Oral Surg Oral Med Oral Pathol Oral Radiol Endod.

[12] Friedlander AH, Sung EC, Chung EM, Garrett NR (2010). Radiographic quantification of chronic dental infection and its relationship to the atherosclerotic process in the carotid arteries. Oral Surg Oral Med Oral Pathol Oral Radiol Endod.

[13] Asai K, Yamori M, Yamazaki T (2015). Tooth loss and atherosclerosis: the Nagahama study. J Dent Res.

[14] Smit MD, Westra J, Vissink A, Doornbos-van der Meer B, Brouwer E, van Winkelhoff AJ (2012). Periodontitis in established rheumatoid arthritis patients: a cross-sectional clinical, microbiological and serological study. Arthritis Res Ther.

[15] Iwasaki M, Taylor GW, Nesse W, Vissink A, Yoshihara A, Miyazaki H (2012). Periodontal disease and decreased kidney function in Japanese elderly. Am J Kidney Dis.

[16] Tada A, Miura H (2012). Prevention of aspiration pneumonia (AP) with oral care. Arch Gerontol Geriatr.

[17] Lexomboon D, Trulsson M, Wårdh I, Parker MG (2012). Chewing ability and tooth loss: association with cognitive impairment in an elderly population study. J Am Geriatr Soc.

[18] Kamer AR, Pirraglia E, Tsui W (2015). Periodontal disease associates with higher brain amyloid load in normal elderly. Neurobiol Aging.

[19] Li H, Takeshita T, Furuta M (2012). Molecular characterization of fungal populations on the tongue dorsum of institutionalized elderly adults. Oral Dis.

[20] Unlüer S, Gökalp S, Doğan BG (2007). Oral health status of the elderly in a residential home in Turkey. Gerodontology.

[21] Zuluaga DJ, Ferreira J, Montoya JA, Willumsen T (2012). Oral health in institutionalised elderly people in Oslo, Norway and its relationship with dependence and cognitive impairment. Gerodontology.

[22] Gerritsen PF, Cune MS, van der Bilt A, de Putter C (2011). Dental treatment needs in Dutch nursing homes offering integrated dental care. Spec Care Dentist.

[23] fVerenso (2015) Richtlijn Mondzorg voor zorgafhankelijke cliënten in verpleeghuizen. http://www.verenso.nl/assets/Uploads/Downloads/Richtlijnen/Richtlijnmondzorg.pdf Accessed October 30 2015

[24] Mombelli A, Gusberti FA, van Oosten MA, Lang NP (1989). Gingival health and gingivitis development during puberty. A 4-year longitudinal study. J Clin Periodontol.

[25] Sectie epidemiologie (2015) Tand en mondverzorging. GGD Groningen http://gemeente.groningen.nl/bsd/nieuws/de-stad-in-cijfers/publicaties/gezondh02-tand-mond.pdf/view Accessed October 30, 2015

[26] Otten F (2003) Steeds minder mensen hebben een kunstgebit. CBS Centraal bureau voor de statistiek (dutch agency for statistics) http://www.cbs.nl/nl-NL/menu/themas/gezondheid-welzijn/publicaties/artikelen/archief/2003/2003-1308-wm.htm Accessed October 30, 2015

[27] Frenkel H, Harvey I, Newcombe RG (2000). Oral health care among nursing home residents in Avon. Gerodontology.

[28] Isaksson R, Söderfeldt B (2007). Oral status and treatment needs among elderly within municipal long-term care 2002–2004. Swed Dent J.

[29] Van der Putten GJ, De Visschere L, Schols J, de Baat C, Vanobbergen J (2012). Supervised versus non-supervised implementation of an oral health care guideline in (residential) care homes: a cluster randomized controlled clinical trial. BMC Oral Health.

[30] Khanagar S, Kumar A, Rajanna V, Badiyani BK, Jathanna VR, Kini PV (2014). Oral health care education and its effect on caregivers’ knowledge, attitudes, and practices: a randomized controlled trial. J Int Soc Prev Community Dent.

[31] Forsell M, Sjögren P, Kullberg E, Johansson O, Wedel P, Herbst B, Hoogstraate J (2011). Attitudes and perceptions towards oral hygiene tasks among geriatric nursing home staff. Int J Dent Hyg.

[32] Han P, Suarez-Durall P, Mulligan R (2015). Dry mouth: a critical topic for older adult patients. J Prosthodont Res.

[33] Anil S, Vellappally S, Hashem M, Preethanath RS, Patil S, Samaranayake LP (2014). Xerostomia in geriatric patients: a burgeoning global concern. J Investig Clin Dent doi.

[34] Visser A, Hoeksema AR, de Baat C, Vissink A (2011). Oral implants in dependent elderly: blessing or burden?. Gerodontology.

[35] Van der Putten GJ, de Baat C, De Visschere L, Schols J (2014). Poor oral health, a potential new geriatric syndrome. Gerodontology.

[36] Cohen-Mansfield J, Thein K, Marx MS, Dakheel-Ali M, Freedman L (2012). Efficacy of non pharmacologic interventions for agitation in advanced dementia: a randomized, placebo-controlled trial. J Clin Psychiatry.

[37] Husebo BS, Ballard C, Sandvik R, Nilsen OB, Aarsland D (2011). Efficacy of treating pain to reduce behavioural disturbances in residents of nursing homes with dementia: cluster randomised clinical trial. BMJ.

